# Supplementary material for: Conjugative Transfer of Acute Hepatopancreatic Necrosis Disease-Causing pVA1-Type Plasmid Is Mediated by a Novel Self-Encoded Type IV Secretion System
Source: Microbiol Spectr. 2022 Sep 19;10(5):e01702-22. doi: 10.1128/spectrum.01702-22 (PMC9602635; doi:10.1128/spectrum.01702-22)
Supplement: Supplemental file 1 — Fig. S1 to S5, Table S1. Download spectrum.01702-22-s0001.pdf, PDF file, 0.7 MB [file spectrum.01702-22-s0001.pdf]

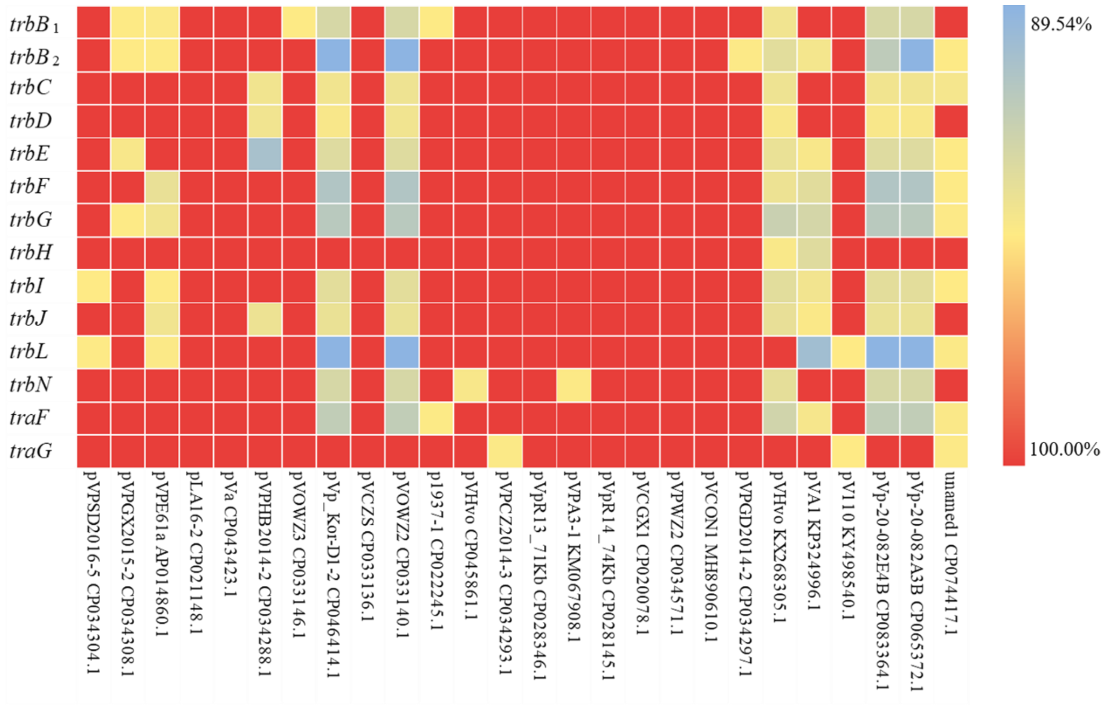

Fig S1. Nucleotide identities of the T4SS components across pVA1-type plasmids against the reference pVPGX1.

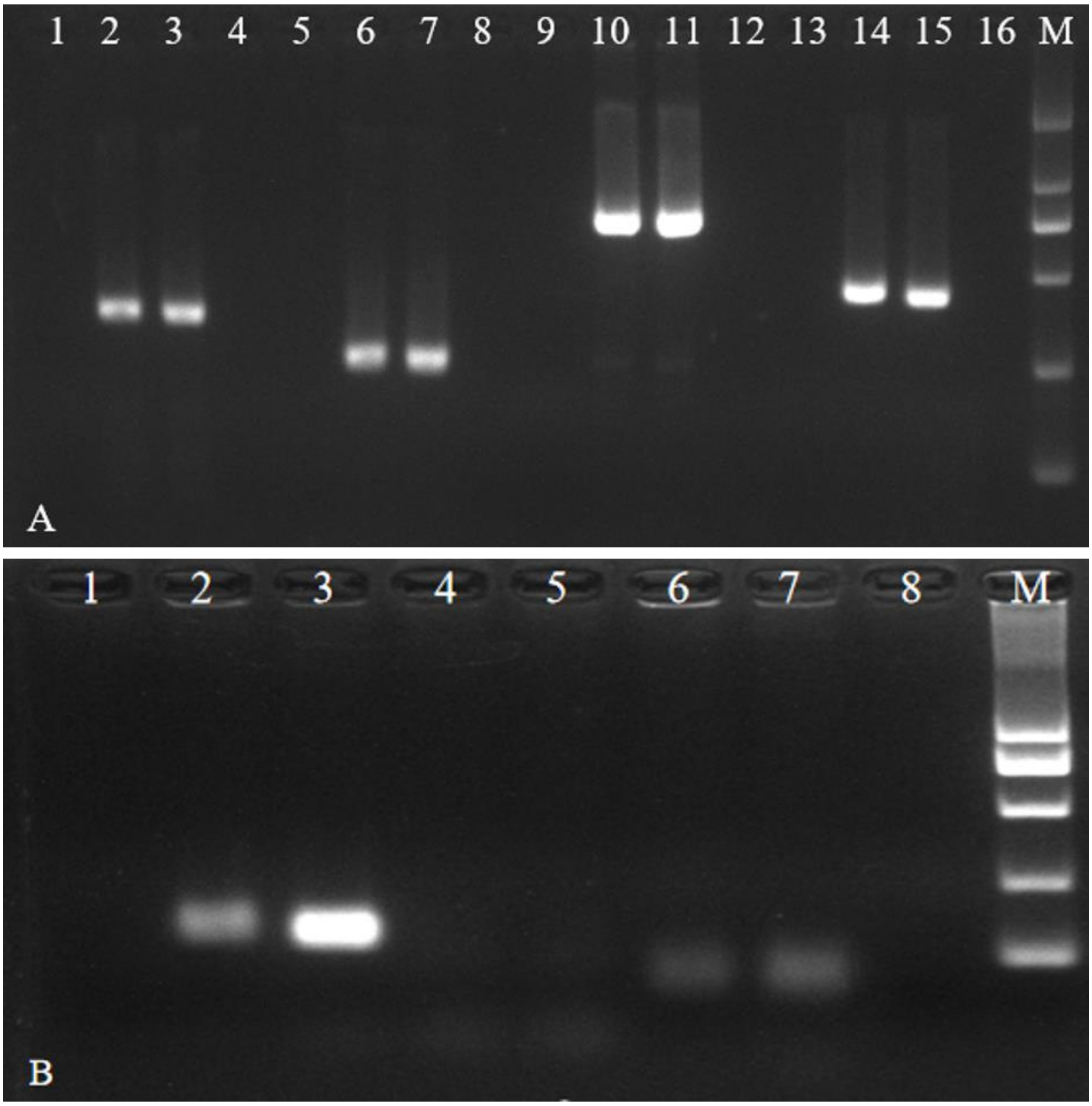

Fig S2. Electrophoretogram of adjacent gene integrity verification of the deletion strains. (A) The PCR results of adjacent gene integrity verification of the deletion strain *Vp*2S01Δ*trbE*, Lanes1-4: the results using the primer pair trbE-del-1F/R, Lanes 5-8: the results using the primer pair trbE-del-2F/R, Lanes 9-12: the results using the primer pair trbE-del-3F/R, Lanes13-16: the results using the primer pair trbE-del-4F/R. Lanes 1, 5, 9, 13: *Vp*2S01Δ*trbE* RNA. Lanes 2, 6, 10, 14: *Vp*2S01Δ*trbE* cDNA. Lanes 3, 7, 11, 15: *Vp*2S01Δ*trbE* DNA. Lanes 4, 8, 12, 16: water (negative control). M: 2Kb Marker. (B) The PCR results of adjacent gene integrity verification of the deletion strain *Vp*2S01Δ*traG*, Lanes1-4: the results using the primer pair traG-del-1F/R, Lanes 5-8: the results using the primer pair traG-del-2F/R, Lanes 1, 5: *Vp*2S01Δ*traG* RNA. Lanes 2, 6: *Vp*2S01Δ*traG* cDNA. Lanes 3, 7: *Vp*2S01Δ*traG* DNA. Lanes 4, 8: water (negative control). M: 2Kb Marker.

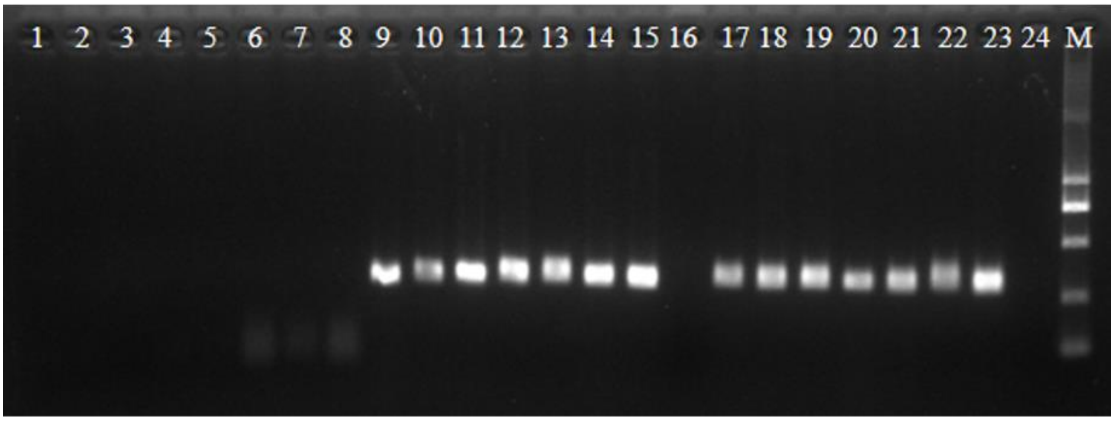

Fig S3. PCR results of the complementation strain *Vp*2S01Δ*trbE*::pRK415-*trbE* using the primer pair trbE-com-F/R. Lanes 1-8: RNA. Lanes 9-16: cDNA. Lanes 17-24: DNA. Lanes 1, 9, 17: the *Vp*2S01Δ*trbE*::pRK415-*trbE* first-generation cultured without tetracycline. Lanes 2, 10, 18: the *Vp*2S01Δ*trbE*::pRK415-*trbE* first-generation cultured with tetracycline. Lanes 3, 11, 19: the *Vp*2S01Δ*trbE*::pRK415-*trbE* second-generation cultured without tetracycline. Lanes 4, 12, 20: the *Vp*2S01Δ*trbE*::pRK415-*trbE* second-generation cultured with tetracycline. Lanes 5, 13, 21: the *Vp*2S01Δ*trbE*::pRK415-*trbE* fifth-generation cultured without tetracycline. Lanes 6, 14, 22: the *Vp*2S01Δ*trbE*::pRK415-*trbE* fifth-generation cultured with tetracycline. Lanes 7, 15, 23: the strain *Vp*2S01::*cat*, Lanes 8, 16, 24:the strain *Vp*2S01Δ*trbE*. M: 2Kb Marker.

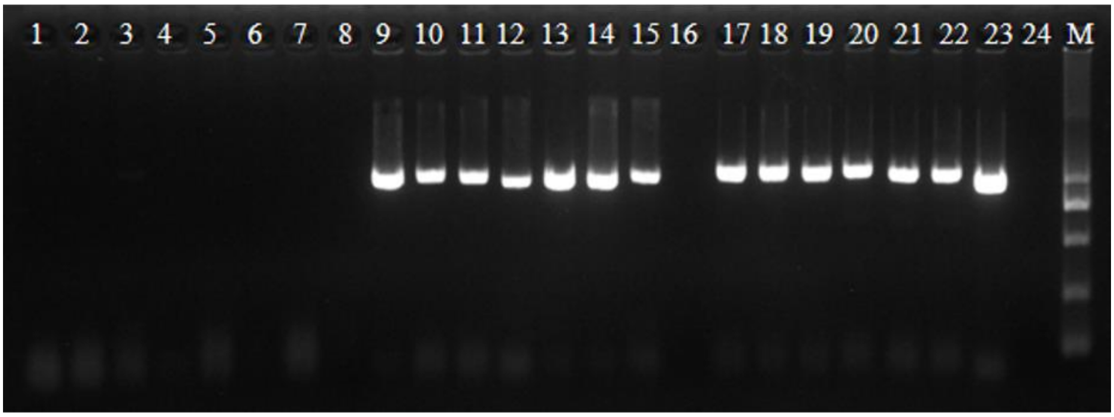

Fig S4. The PCR results of the complementation strain *Vp*2S01Δ*traG*::pRK415-*traG* using the primer pair traG-com-F/R. Lanes 1-8: RNA. Lanes 9-16: cDNA. Lanes 17-24: DNA. Lanes 1, 9, 17: the *Vp*2S01Δ*traG*::pRK415-*traG* first-generation cultured without tetracycline. Lanes 2, 10, 18: the *Vp*2S01Δ*traG*::pRK415-*traG* first-generation cultured with tetracycline. Lanes 3, 11, 19: the *Vp*2S01Δ*traG*::pRK415-*traG* second-generation cultured without tetracycline. Lanes 4, 12, 20: the *Vp*2S01Δ*traG*::pRK415-*traG* second-generation cultured with tetracycline. Lanes 5, 13, 21: the *Vp*2S01Δ*traG*::pRK415-*traG* fifth-generation cultured without tetracycline. Lanes 6, 14, 22: the *Vp*2S01Δ*traG*::pRK415-*traG* fifth-generation cultured with tetracycline. Lanes 7, 15, 23: the strain *Vp*2S01::*cat*, Lanes 8, 16, 24:the strain *Vp*2S01Δ*traG*. M: 2Kb Marker.

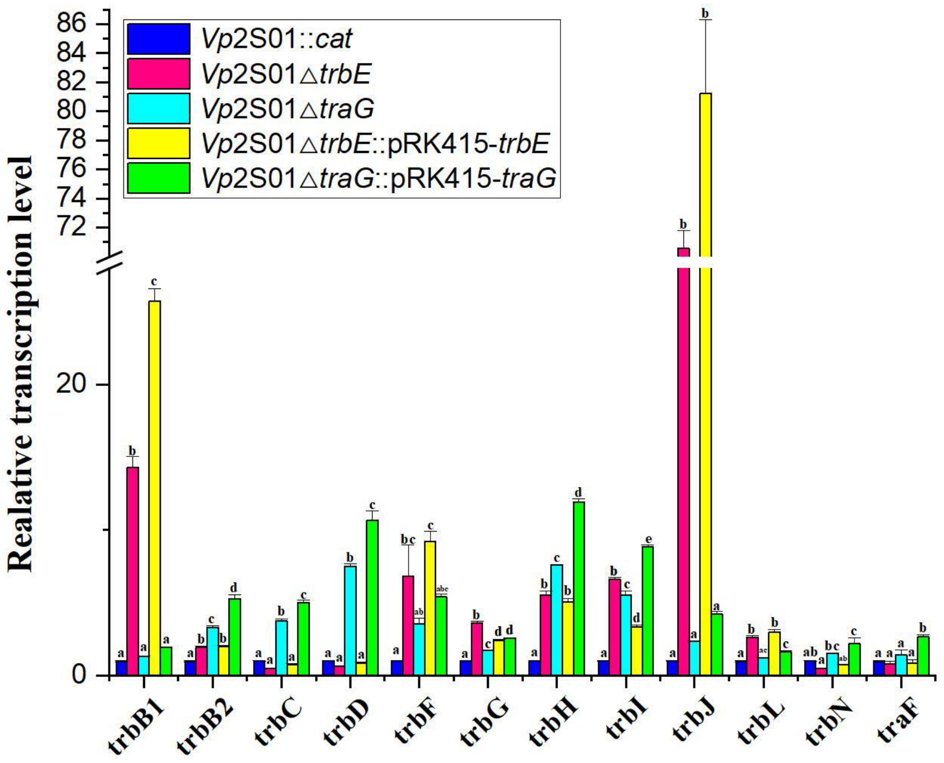

Fig S5. RT-qPCR analysis of expression of T4SS genes in strains, including *Vp*2S01::*cat*, *Vp*2S01Δ*trbE*, *Vp*2S01Δ*traG*, *Vp*2S01Δ*trbE*::pRK415-*trbE*, and *Vp*2S01Δ*traG*::pRK415-*traG*. Each value represents the mean ± standard deviation (*SD*). Statistical analysis was performed by One-Way ANOVA. The same lower case letters on each row indicate no significant difference, and different lower case letters on each line indicate a significant difference ($P < 0.05$). All the experiments were repeated thrice.

Table S1. Reference sequences of T4SS for phylogenetic analysis.

#	Classification Scheme 1	Classification Scheme 2	T4SS name	Bacteria name or plasmid name	Reference sequences in NCBI		
					TraD-F	TraG-F	TraC-F
1	Type IVA	Type F	Tra_F	*Acidithiobacillus ferrivorans* SS3	WP_014030300.1	WP_014030287.1	WP_014030354.1
2	Type IVA	Type F	Tra_F	*Acidovorax* sp. JS42	WP_011804822.1	WP_011804797.1	WP_011804808.1
3	Type IVA	Type F	Tra_F	*Burkholderia glumae* BGR1	WP_012735302.1	WP_012735277.1	WP_012735290.1
4	Type IVA	Type F	Tra_F	*Candidatus Protochlamydia amoebophila* UWE25	WP_011175990.1	WP_011175989.1	WP_011175979.1
5	Type IVA	Type F	Tra_F	*Dechloromonas aromatica* RCB	WP_011288042.1	WP_011288066.1	WP_011288055.1
6	Type IVA	Type F	Tra_F	*Enterobacter asburiae* LF7a	WP_014063900.1	WP_014063895.1	WP_014063876.1
7	Type IVA	Type F	Tra_F	*Enterobacter* sp. 638	WP_011906581.1	WP_011906577.1	WP_011906563.1
8	Type IVA	Type F	Tra_F	*Escherichia coli* 1520 plasmid pIP1206	WP_012372807.1	WP_001553826.1	WP_001064264.1
9	Type IVA	Type F	Tra_F	*Escherichia coli* plasmid pETN48	WP_013362804.1	WP_013362803.1	WP_001064245.1
10	Type IVA	Type F	Tra_F	*Escherichia coli* S88	WP_001350920.1	WP_012601910.1	WP_001064252.1
11	Type IVA	Type F	Tra_F	*Klebsiella pneumoniae* plasmid pK29	WP_001284073.1	WP_000386160.1	WP_000387412.1
12	Type IVA	Type F	Tra_F	*Legionella pneumophila* str. Lens	WP_011212621.1	WP_011212619.1	WP_011212610.1
13	Type IVA	Type F	Tra_F	*Orientia tsutsugamushi* str. Ikeda	WP_012462245.1	WP_012462243.1	WP_012462234.1
14	Type IVA	Type F	Tra_F	*Photobacterium damselae* subsp. piscicida plasmid pP91278	WP_000119468.1	WP_000534551.1	WP_000637386.1
15	Type IVA	Type F	Tra_F	*Rickettsia bellii* RML369-C	WP_008579926.1	WP_008579923.1	WP_011477112.1
16	Type IVA	Type F	Tra_F	*Salmonella enterica* subsp. enterica serovar Typhi plasmid R27	WP_000167420.1	WP_010892329.1	WP_001255540.1

17	Type IVA	Type F	Tra_F	*Yersinia pestis biovar Orientalis* str. IP275 plasmid pIP1202	WP_000178856.1	WP_000534551.1	WP_000637386.1
					VirD4	VirB6	VirB4
18	Type IVA	Type P	VirB	*Acidiphilium cryptum JF-5* plasmid pACRY03	WP_011930797.1	WP_011930791.1	WP_011930789.1
19	Type IVA	Type P	VirB	*Aeromonas caviae* plasmid pFBAOT6	WP_011191320.1	WP_011191326.1	WP_011191329.1
20	Type IVA	Type P	VirB	*Agrobacterium radiobacter* K84	WP_012652936.1	WP_007694690.1	WP_012652920.1
21	Type IVA	Type P	VirB	*Agrobacterium radiobacter* K84	WP_010900359.1	WP_010900347.1	WP_010900345.1
22	Type IVA	Type P	VirB	*Agrobacterium tumefaciens* plasmid Ti plasmid pTiBo542	WP_012478088.1	WP_012478076.1	WP_012478074.1
23	Type IVA	Type P	VirB	*Bartonella clarridgeiae* 73	WP_013544966.1	WP_013544433.1	WP_013544435.1
24	Type IVA	Type P	VirB	*Bartonella quintana* str. Toulouse	WP_011179734.1	WP_011179728.1	WP_011179726.1
25	Type IVA	Type P	VirB	*Burkholderia cenocepacia* AU 1054	WP_011548469.1	WP_011548475.1	WP_011548476.1
26	Type IVA	Type P	VirB	*Campylobacter lari* RM2100	WP_012662270.1	WP_012662263.1	WP_012662258.1
27	Type IVA	Type P	VirB	*Dickeya dadantii* Ech586	WP_012884152.1	WP_012884158.1	WP_012884160.1
28	Type IVA	Type P	VirB	*Edwardsiella tarda EIB202* plasmid pEIB202	WP_012850347.1	WP_012850355.1	WP_012850359.1
29	Type IVA	Type P	VirB	*Gluconacetobacter xylinus* NBRC 3288 plasmid pGXY020	WP_007284439.1	WP_007284444.1	WP_007284447.1
30	Type IVA	Type P	VirB	*Mesorhizobium loti* MAFF303099	WP_010915832.1	WP_010915837.1	WP_010915840.1
31	Type IVA	Type P	VirB	*Nitrobacter hamburgensis* X14	WP_011505126.1	WP_011505121.1	WP_011505117.1
32	Type IVA	Type P	VirB	*Oligotropha carboxidovorans OM5* plasmid pOC167	WP_012564827.1	WP_012564822.1	WP_012564817.1
33	Type IVA	Type P	VirB	*Rhizobium leguminosarum* bv. trifolii WSM2304	WP_012559858.1	WP_012559853.1	WP_012559849.1
34	Type IVA	Type P	VirB	*Yersinia pestis biovar Microtus* str. 91001 plasmid pCRY	WP_011172019.1	WP_011172012.1	WP_011172009.1
					TraG	TrbL	TrbE
35	Type IVA	Type P	Trb	*Birmingham IncP-alpha* plasmid	YP_001687707.1	YP_001687681.1	YP_001687674.1
36	Type IVA	Type P	Trb	*uncultured bacterium* plasmid pTB11	WP_011205817.1	WP_011205794.1	WP_011205787.1

37	Type IVA	Type P	Trb	*Legionella pneumophila* 2300/99 Alcoy	WP_011945356.1	WP_013100980.1	WP_011945348.1
38	Type IVA	Type P	Trb	*Neisseria gonorrhoeae* plasmid pEP5289	WP_003701841.1	WP_003701894.1	WP_003701881.1
39	Type IVA	Type P	Trb	*Legionella longbeachae* NSW150	WP_012979416.1	WP_012979415.1	WP_012979408.1
40	Type IVA	Type P	Trb	*Burkholderia cepacia* plasmid pIJB1	YP_003358106.1	YP_003358081.1	YP_003358074.1
41	Type IVA	Type P	Trb	*Photobacterium damselae* subsp. Piscicida plasmid pP9014	WP_012774826.1	WP_012774856.1	WP_012774849.1
42	Type IVA	Type P	Trb	*Comamonas testosteroni* CNB-1	WP_012478237.1	WP_012478193.1	WP_011013264.1
43	Type IVA	Type P	Trb	*Xylella fastidiosa* M23	WP_004086666.1	WP_004086703.1	WP_004086690.1
44	Type IVA	Type P	Trb	*Yersinia pseudotuberculosis* IP 31758	WP_011988415.1	WP_011988447.1	WP_011988420.1
45	Type IVA	Type P	Trb	*uncultured bacterium* plasmid pB4	WP_011013286.1	WP_011013271.1	WP_011013264.1
46	Type IVA	Type P	Trb	*uncultured bacterium* plasmid pB10	WP_000694765.1	WP_011114051.1	WP_011114047.1
47	Type IVA	Type P	Trb	*Pseudomonas* sp. ADP plasmid pADP-1	WP_000694765.1	WP_011114051.1	WP_011114047.1
48	Type IVA	Type P	Trb	Plasmid pB3	WP_011222099.1	WP_011222086.1	WP_011222080.1
49	Type IVA	Type P	Trb	*Aromatoleum aromaticum* EbN1	WP_011255047.1	WP_011255073.1	WP_011255080.1
50	Type IVA	Type P	Trb	*Achromobacter xylosoxidans* A8	WP_011255185.1	WP_011255137.1	WP_011255132.1
51	Type IVA	Type P	Trb	*Ralstonia eutropha* JMP134	WP_000694765.1	WP_011114051.1	WP_011114047.1
52	Type IVA	Type P	Trb	*Sphingomonas* sp. A1 plasmid pA1	WP_011304093.1	WP_011304126.1	WP_011304119.1
53	Type IVA	Type P	Trb	*IncP-1beta multiresistance* plasmid pB8	WP_000694765.1	WP_011114051.1	WP_011342945.1
54	Type IVA	Type P	Trb	*uncultured bacterium* plasmid pTP6	WP_000694765.1	WP_011114051.1	WP_011114047.1
55	Type IVA	Type P	Trb	Plasmid QKH54	WP_011544437.1	WP_011544414.1	WP_011544407.1
56	Type IVA	Type P	Trb	*IncP-1* plasmid pKJK5	WP_011600635.1	WP_011600621.1	WP_011600613.1
57	Type IVA	Type P	Trb	*Bordetella pertussis* plasmid pBP136	WP_011666400.1	WP_011666389.1	WP_011666383.1
58	Type IVA	Type P	Trb	*Legionella pneumophila* str. Corby	WP_011945356.1	WP_011945355.1	WP_011945348.1

59	Type IVA	Type P	Trb	*Nitrosomonas eutropha* C91	WP_011630654.1	WP_011630634.1	WP_011630636.1
60	Type IVA	Type P	Trb	*Rhizobium leguminosarum* bv. trifolii WSM1325	WP_012760272.1	WP_012760244.1	WP_012760241.1
61	Type IVA	Type P	Trb	*Acidovorax* sp. JS42	WP_011806065.1	WP_011805731.1	WP_011806060.1
62	Type IVA	Type P	Trb	*Pseudomonas aeruginosa* UCBPP-PA14	WP_003138988.1	WP_003138975.1	WP_003138981.1
63	Type IVA	Type P	Trb	*Photobacterium profundum* SS9	WP_011221638.1	WP_011221619.1	WP_011221616.1
64	Type IVA	Type P	Trb	*Bradyrhizobium japonicum* USDA 110	WP_011082875.1	WP_011090990.1	WP_011090993.1
65	Type IVA	Type P	Trb	*Oligotropha carboxidovorans* OM5	WP_013913800.1	WP_013913815.1	WP_013913818.1
66	Type IVA	Type P	Trb	*Nitrobacter hamburgensis* X14	WP_011505305.1	WP_011505218.1	WP_011505221.1
67	Type IVA	Type P	Trb	*Rhizobium leguminosarum* bv. viciae 3841	WP_011654742.1	WP_011654758.1	WP_011654760.1
68	Type IVA	Type P	Trb	*Azorhizobium caulinodans* ORS 571	WP_012172348.1	WP_012172384.1	WP_012172381.1
69	Type IVA	Type P	Trb	*Methylobacterium populi* BJ001	WP_012454957.1	WP_012454965.1	WP_012454962.1
70	Type IVA	Type P	Trb	*Sinorhizobium meliloti* SM11 plasmid pSmeSM11b	WP_012477334.1	WP_012477353.1	WP_012477356.1
71	Type IVA	Type P	Trb	*Agrobacterium tumefaciens* plasmid Ti plasmid pTiBo542	WP_012478134.1	WP_012478019.1	WP_012478022.1
72	Type IVA	Type P	Trb	*Agrobacterium radiobacter* K84	WP_012654905.1	WP_012654965.1	WP_012654968.1
73	Type IVA	Type P	Trb	*Sinorhizobium fredii* NGR234	WP_010875407.1	WP_010875424.1	WP_010875427.1
74	Type IVA	Type P	Trb	*Sinorhizobium medicae* WSM419	WP_011970273.1	WP_011970260.1	WP_011970257.1
75	Type IVA	Type P	Trb	*Gluconacetobacter diazotrophicus* PAl 5	WP_012553468.1	WP_012553461.1	WP_012553463.1
76	Type IVA	Type P	Trb	*Cupriavidus metallidurans* CH34	WP_011516287.1	WP_011516293.1	WP_011516290.1
77	Type IVA	Type P	Trb	*Thiomonas intermedia* K12	WP_013123563.1	WP_013123553.1	WP_013123556.1
78	Type IVA	Type P	Trb	*Halothiobacillus neapolitanus* c2	WP_012824509.1	WP_012824476.1	WP_012824479.1
79	Type IVA	Type P	Trb	*Caulobacter* sp. K31	WP_012286064.1	WP_012286071.1	WP_012286069.1
80	Type IVA	Type P	Trb	*Xanthobacter autotrophicus* Py2	WP_012114865.1	WP_012114873.1	WP_012114870.1

81	Type IVA	Type P	Trb	*Gluconacetobacter diazotrophicus* PAl 5	WP_012222371.1	WP_012222378.1	WP_012222376.1
82	Type IVA	Type P	Trb	*Mesorhizobium loti* MAFF303099	WP_010916057.1	WP_010916175.1	WP_010916173.1
83	Type IVA	Type P	Trb	*Rhodopseudomonas palustris* BisA53	WP_011665412.1	WP_011665404.1	WP_011665407.1
84	Type IVA	Type P	Trb	*Stenotrophomonas maltophilia* K279a	WP_012479466.1	WP_012479459.1	WP_012479461.1
85	Type IVA	Type P	Trb	*Hirschia baltica ATCC* 49814	WP_015826522.1	WP_015826513.1	WP_015826515.1
86	Type IVA	Type P	Trb	*Caulobacter* sp. K31	WP_012279436.1	WP_012279493.1	WP_012279497.1
87	Type IVA	Type P	Trb	*Sphingobium japonicum* UT26S	WP_013041717.1	WP_013041709.1	WP_013041712.1
88	Type IVA	Type P	Trb	*Azotobacter vinelandii* DJ	WP_012702100.1	WP_012702092.1	WP_012702095.1
89	Type IVA	Type P	Trb	*Ralstonia solanacearum* GMI1000	WP_011002501.1	WP_011002493.1	WP_011002496.1
90	Type IVA	Type P	Trb	*Rhodopseudomonas palustris* BisB18	WP_011473974.1	WP_011473961.1	WP_011473964.1
91	Type IVA	Type P	Trb	*Burkholderia ambifaria* AMMD	WP_011657243.1	WP_011657251.1	WP_011657248.1
92	Type IVA	Type P	Trb	*Shewanella baltica* OS195	WP_012198033.1	WP_012198012.1	WP_012198010.1
93	Type IVA	Type P	Trb	*Prosthecochloris aestuarii* DSM 271	WP_012509540.1	WP_012509548.1	WP_012509545.1
94	Type IVA	Type P	Trb	*Thioalkalivibrio sulfidophilus* HL-EbGr7	WP_012638455.1	WP_012638461.1	WP_012638459.1
95	Type IVA	Type P	Trb	*Desulfovibrio salexigens* DSM 2638	WP_015851566.1	WP_015851582.1	WP_015851584.1
96	Type IVA	Type P	Trb	*Sphingobium japonicum* UT26S	WP_013041098.1	WP_013041091.1	WP_013041093.1
97	Type IVA	Type P	Trb	*Burkholderia multivorans* ATCC 17616	WP_006414886.1	WP_012213896.1	WP_006414878.1
98	Type IVA	Type P	Trb	*Polaromonas naphthalenivorans* CJ2	WP_011797849.1	WP_011797856.1	WP_011797854.1
99	Type IVA	Type P	Trb	*Phenylobacterium zucineum* HLK1	WP_012522063.1	WP_012522071.1	WP_012522069.1
100	Type IVA	Type P	Trb	*Pseudomonas aeruginosa* PA7	WP_012076309.1	WP_012076302.1	WP_012076305.1
101	Type IVA	Type P	Trb	*Ralstonia pickettii* 12J	WP_012436231.1	WP_012436238.1	WP_012436236.1
102	Type IVA	Type P	Trb	*Rhodopseudomonas palustris* CGA009-1	WP_011157788.1	WP_011157780.1	WP_011157783.1

103	Type IVA	Type P	Trb	*Rhodopseudomonas palustris* CGA009-2	WP_011159667.1	WP_011159653.1	WP_011159656.1
104	Type IVA	Type P	Trb	*Bradyrhizobium* sp. BTAi1	WP_012041749.1	WP_012041761.1	WP_012041759.1
105	Type IVA	Type P	Trb	*Acidiphilium multivorum* AIU301	WP_013639391.1	WP_013639399.1	WP_013639396.1
106	Type IVA	Type P	Trb	*Delftia acidovorans* SPH-1	WP_012202376.1	WP_012202369.1	WP_012202372.1
107	Type IVA	Type P	Trb	*Oligotropha carboxidovorans* OM5	WP_012564734.1	WP_012564726.1	WP_012564729.1
108	Type IVA	Type P	Trb	*Bordetella petrii* DSM 12804	WP_012249115.1	WP_012249123.1	WP_012249120.1
109	Type IVA	Type P	Trb	*Mesorhizobium opportunistum* WSM2075	WP_013893940.1	WP_013893993.1	WP_013893989.1
110	Type IVA	Type P	Trb	*Parvularcula bermudensis* HTCC2503	WP_013300181.1	WP_013300190.1	WP_013300188.1
111	Type IVA	Type P	Trb	*Acidovorax ebreus* TPSY	WP_015912941.1	WP_015912947.1	WP_015912944.1
112	Type IVA	Type P	Trb	*Oligotropha carboxidovorans* OM5	WP_012564734.1	WP_012564726.1	WP_012564729.1
113	Type IVA	Type P	Trb	*Desulfotalea psychrophila* LSv54	WP_011190383.1	WP_011190375.1	WP_011190373.1
114	Type IVA	Type P	Trb	*Methylovorus glucosetrophus* SIP3-4	WP_012777702.1	WP_012777725.1	WP_012777727.1
115	Type IVA	Type P	Trb	*Zymomonas mobilis* subsp. mobilis NCIMB 11163	WP_015740162.1	WP_015740168.1	WP_015740166.1
116	Type IVA	Type P	Trb	*Acidithiobacillus ferrooxidans* ATCC 53993	WP_012536711.1	WP_012536665.1	WP_012535917.1
117	Type IVA	Type P	Trb	*Caulobacter segnis* ATCC 21756	WP_013079659.1	WP_013079605.1	WP_013079608.1
118	Type IVA	Type P	Trb	*Acidithiobacillus ferrooxidans* ATCC 23270	WP_012607179.1	WP_012607152.1	WP_012607148.1
119	Type IVA	Type P	Trb	*Achromobacter xylosoxidans* A8	WP_012478237.1	WP_012478193.1	WP_011013264.1
120	Type IVA	Type P	Trb	*Acidovorax* sp. JS42	WP_011798733.1	WP_011798700.1	WP_011798706.1
121	Type IVA	Type P	Trb	*Agrobacterium rhizogenes*	WP_010900298.1	WP_010900313.1	WP_010900316.1
122	Type IVA	Type P	Trb	*Agrobacterium rhizogenes*	WP_012475987.1	WP_012476001.1	WP_012476004.1
123	Type IVA	Type P	Trb	*Agrobacterium tumefaciens*	WP_010891537.1	WP_010891630.1	WP_010891627.1
124	Type IVA	Type P	Trb	*Agrobacterium tumefaciens* plasmid Ti	WP_010892380.1	WP_010892442.1	WP_010892445.1

125	Type IVA	Type P	Trb	*Agrobacterium tumefaciens* str. C58	WP_010974894.1	WP_010974835.1	WP_010974837.1
126	Type IVA	Type P	Trb	*Agrobacterium vitis* S4	WP_012648972.1	WP_012649096.1	WP_012649099.1
127	Type IVA	Type P	Trb	*Agrobacterium vitis* S4	WP_012655076.1	WP_012655115.1	WP_012655117.1
128	Type IVA	Type P	Trb	*Agrobacterium vitis* S4	WP_012650583.1	WP_012650635.1	WP_012650638.1
129	Type IVA	Type P	Trb	*Burkholderia ambifaria* AMMD	WP_011655356.1	WP_011114051.1	WP_011114047.1
130	Type IVA	Type P	Trb	*Burkholderia gladioli* BSR3	WP_013698452.1	WP_013698446.1	WP_013698448.1
131	Type IVA	Type P	Trb	*Burkholderia pseudomallei* MSHR346	WP_012729957.1	WP_012730033.1	WP_012730359.1
132	Type IVA	Type P	Trb	*Candidatus Accumulibacter phosphatis clade IIA* str. UW-1	WP_012806753.1	WP_012806821.1	WP_012806827.1
133	Type IVA	Type P	Trb	*Chelativorans* sp. BNC1	WP_011579861.1	WP_011579849.1	WP_011579852.1
134	Type IVA	Type P	Trb	*Delftia acidovorans* plasmid pUO1	WP_000694765.1	WP_011114051.1	WP_011114047.1
135	Type IVA	Type P	Trb	*Dickeya dadantii* 3937	WP_013316517.1	WP_013316524.1	WP_013316522.1
136	Type IVA	Type P	Trb	*Enterobacter aerogenes*	WP_010890148.1	WP_010890137.1	WP_010890130.1
137	Type IVA	Type P	Trb	*Ketogulonicigenium vulgare* Y25	WP_013368406.1	WP_013368414.1	WP_013368411.1
138	Type IVA	Type P	Trb	*Mesorhizobium ciceri biovar biserrulae* WSM1271	WP_013533272.1	WP_013533265.1	WP_013533267.1
139	Type IVA	Type P	Trb	*Mesorhizobium loti* MAFF303099	WP_010914016.1	WP_010914022.1	WP_010914021.1
140	Type IVA	Type P	Trb	*Nitrosomonas* sp. AL212 plasmid pNAL21201	WP_013648939.1	WP_013648912.1	WP_013648919.1
141	Type IVA	Type P	Trb	*Nitrosomonas* sp. AL212 plasmid pNAL21202	WP_013646170.1	WP_013646142.1	WP_013646149.1
142	Type IVA	Type P	Trb	*Novosphingobium aromaticivorans* DSM 12444	WP_011443982.1	WP_011443974.1	WP_011443977.1
143	Type IVA	Type P	Trb	*Novosphingobium* sp. PP1Y	WP_013831682.1	WP_013831696.1	WP_013831694.1
144	Type IVA	Type P	Trb	*Ochrobactrum anthropi* ATCC 49188	WP_012092636.1	WP_012092661.1	WP_012092658.1
145	Type IVA	Type P	Trb	*Paracoccus denitrificans* PD1222	WP_011749455.1	WP_011749466.1	WP_011749464.1
146	Type IVA	Type P	Trb	*Paracoccus denitrificans* PD1222	WP_011746492.1	WP_011746500.1	WP_011746503.1

147	Type IVA	Type P	Trb	*Parvibaculum lavamentivorans* DS-1	WP_012110743.1	WP_012110750.1	WP_012110748.1
148	Type IVA	Type P	Trb	*Polaromonas naphthalenivorans* CJ2	WP_011801911.1	WP_011801887.1	WP_011801885.1
149	Type IVA	Type P	Trb	*Pseudomonas mendocina* NK-01	WP_013716001.1	WP_013716009.1	WP_013716006.1
150	Type IVA	Type P	Trb	*Ralstonia eutropha* JMP134 plasmid pJP4	WP_000694765.1	WP_011114051.1	WP_011114047.1
151	Type IVA	Type P	Trb	*Rhizobium etli* CFN 42	WP_011427440.1	WP_011427457.1	WP_011427459.1
152	Type IVA	Type P	Trb	*Shewanella* sp. ANA-3	WP_011716356.1	WP_011716364.1	WP_011716361.1
153	Type IVA	Type P	Trb	*Sphingobium* sp. SYK-6	WP_014076927.1	WP_014076920.1	WP_014076922.1
154	Type IVA	Type P	Trb	*Sphingomonas wittichii* RW1	WP_012049874.1	WP_012049882.1	WP_012049879.1
155	Type IVA	Type P	Trb	*Sphingopyxis alaskensis* RB2256	WP_011542762.1	WP_011542752.1	WP_011542755.1
156	Type IVA	Type P	Trb	*Vibrio parahaemolyticus* 20130629002S01 plasmid pVPGX1	WP_032072614.1	WP_025789130.1	WP_025789746.1
157	Type IVA	Type P	Trb	*Vibrio parahaemolyticus* 20160303005-1 plasmid pVPSD2016-5	QHH13387.1	QHH13360.1	QHH13357.1
158	Type IVA	Type P	Trb	*Vibrio parahaemolyticus* 20140829008-1 plasmid pVPGD2014-2	QHH07838.1	QHH07793.1	QHH07796.1
159	Type IVA	Type P	Trb	*Vibrio parahaemolyticus* 20140722001-1 plasmid pVPCZ2014-3	QHH02754.1	QHH02778.1	QHH02781.1
160	Type IVA	Type P	Trb	*Vibrio parahaemolyticus* 20140624012-1 plasmid pVPHB2014-2	QHG97597.1	QHG97637.1	QHG97634.1
161	Type IVA	Type P	Trb	*Vibrio parahaemolyticus* MVP1 plasmid pVa	QEL43613.1	QEL43639.1	QEL43642.1
162	Type IVA	Type P	Trb	*Vibrio owensii* V180403 plasmid pVOWZ3	AYO24115.1	AYO24117.1	AYO24144.1
163	Type IVA	Type P	Trb	*Vibrio campbellii* 170502 plasmid pVCZS	AYO12917.1	AYO12944.1	AYO12947.1
164	Type IVA	Type P	Trb	*Vibrio parahaemolyticus* 160807 plasmid pVPWZ2	AZU96078.1	AZU96081.1	AZU96038.1
165	Type IVA	Type P	Trb	*Vibrio parahaemolyticus* PB1937 plasmid p1937-1	WP_032072614.1	WP_025789130.1	WP_025789746.1
166	Type IVA	Type P	Trb	*Vibrio parahaemolyticus* R13 plasmid pVpR13_71Kb	AWJ81933.1	AWJ81961.1	AWJ81964.1
167	Type IVA	Type P	Trb	*Vibrio parahaemolyticus* R14 plasmid pVpR14_74Kb	AWG82335.1	AWG82307.1	AWG82304.1
168	Type IVA	Type P	Trb	*Vibrio parahaemolyticus* plasmid pVPE61a	BAX56889.1	BAX56940.1	BAX56947.1

169	Type IVA	Type P	Trb	*Vibrio campbellii* LA16-V1 plasmid pLA16-2	ARR47955.1	ARR47981.1	ARR47984.1
170	Type IVA	Type P	Trb	*Vibrio campbellii* 20130629003S01 plasmid pVCGX1	ARR10075.1	ARR10109.1	ARR10112.1
171	Type IVA	Type P	Trb	*Vibrio parahaemolyticus* v110 plasmid pV110	AQZ36731.1	AQZ36765.1	AQZ36768.1
172	Type IVA	Type P	Trb	*Vibrio parahaemolyticus* 3HP plasmid pVA1	AKC05643.1	AKC05699.1	AKC05696.1
173	Type IVA	Type P	Trb	*Vibrio parahaemolyticus* 13-028/A3 plasmid pVPA3-1	AIL49888.1	AIL49920.1	AIL49923.1
174	Type IVA	Type P	Trb	*Vibrio parahaemolyticus* 19-021-D1 plasmid pVp_Kor-D1-2	QGT94655.1	QGT94629.1	QGT94626.1
175	Type IVA	Type P	Trb	*Vibrio owensii* 1700302 plasmid pVOWZ2	AYO18592.1	AYO18618.1	AYO18621.1
176	Type IVA	Type P	Trb	*Vibrio owensii* SH14 plasmid pVHvo	QGH51074.1	QGH51071.1	QGH51074.1
177	Type IVA	Type P	Trb	*Vibrio owensii* strain SH14 plasmid pVHvo	AQT24327.1	AQT24361.1	AQT24364.1
178	Type IVA	Type P	Trb	*Vibrio parahaemolyticus* 20-082E4 plasmid pVp-20-082E4B	UAY45907.1	UAY45884.1	UAY45881.1
179	Type IVA	Type P	Trb	*Vibrio parahaemolyticus* 20-082A3 plasmid pVp-20-082A3B	QPM88572.1	QPM88597.1	QPM88600.1
180	Type IVA	Type P	Trb	*Vibrio parahaemolyticus* 64 plasmid unnamed1	CP074417.1	CP074417.1	CP074417.1
181	Type IVA	Type P	Trb	*Vibrio parahaemolyticus* 20151116002-3 plasmid pVPGX2015-2	QHH18394.1	QHH18369.1	WP_017449281.1
182	Type IVA	Type P	Trb	*Vibrio campbellii* strain LMB29 plasmid pVCON1	WP_032072614.1	WP_025789130.1	WP_025789746.1
					TrwB	TrwI	TrwK
183	Type IVA	Type P	Trw	*Anaeromyxobacter dehalogenans* 2CP-1	WP_012632449.1	WP_012632477.1	WP_012632474.1
184	Type IVA	Type P	Trw	*Bordetella petrii* DSM 12804	WP_012250618.1	WP_012250608.1	WP_012250604.1
185	Type IVA	Type P	Trw	*Escherichia coli* plasmid IncN plasmid N3	WP_001749976.1	WP_001749958.1	WP_001749961.1
186	Type IVA	Type P	Trw	*Salmonella enterica* subsp. enterica serovar Dublin plasmid pMAK3	WP_012196433.1	WP_012196426.1	WP_012196423.1
187	Type IVA	Type P	Trw	*Pusillimonas* sp. T7-7	WP_013741385.1	WP_013741404.1	WP_013741407.1
188	Type IVA	Type P	Trw	*Providencia rettgeri* plasmid R7K	WP_012414159.1	WP_012414166.1	WP_012414168.1
189	Type IVA	Type P	Trw	*IncN* plasmid R46	WP_000342688.1	WP_010999960.1	WP_010999957.1

190	Type IVA	Type P	Trw	*Klebsiella oxytoca KOX105* plasmid pKOX105	WP_000342688.1	WP_001749958.1	WP_001749961.1
					LvhD4	LvhB6	LvhB4
191	Type IVA	Type P	Lvh	*Legionella pneumophila* str. Lens	WP_011214439.1	WP_011214434.1	WP_011214431.1
192	Type IVA	Type P	Lvh	*Legionella pneumophila* str. Paris	WP_011214439.1	WP_011214434.1	WP_011214431.1
193	Type IVA	Type P	Lvh	*Legionella pneumophila* subsp. *pneumophila* str. Philadelphia 1	WP_010946977.1	WP_010946982.1	WP_010946986.1
					Cagbeta	CagW	CagE
194	Type IVA	Type P	Cag	*Helicobacter pylori* 26695	WP_000389328.1	WP_000481884.1	WP_000495985.1
195	Type IVA	Type P	Cag	*Helicobacter pylori* G27	WP_000389376.1	WP_000481932.1	WP_000495951.1
196	Type IVA	Type P	Cag	*Helicobacter pylori* J99	WP_000389361.1	WP_000481893.1	WP_000496009.1
197	Type IVA	Type P	Cag	*Helicobacter pylori* P12	WP_000389325.1	WP_000481900.1	WP_000495947.1
198	Type IVA	Type P	Cag	*Helicobacter pylori* Shi470	WP_000389316.1	WP_000481879.1	WP_012443370.1
					MagB12	MagB06	MagB03
199	Type IVA	Type P	MagB	*Aggregatibacter actinomycetemcomitans* D11S-1	WP_013386454.1	WP_013386460.1	WP_013386462.1
200	Type IVA	Type P	MagB	*Aggregatibacter actinomycetemcomitans* plasmid pVT745	WP_010901931.1	WP_010901937.1	WP_010901939.1
					TrbC-I	TraY_I	TraU-I
201	Type IVB	Type I	Tra_I	*Candidatus Hamiltonella defensa* 5AT	WP_012738252.1	WP_012738258.1	WP_015873076.1
202	Type IVB	Type I	Tra_I	*Citrobacter freundii* plasmid pCTX-M3	WP_011091092.1	WP_004187492.1	WP_011091081.1
203	Type IVB	Type I	Tra_I	*Escherichia coli* SE11	WP_001289281.1	WP_000075186.1	WP_001024774.1
204	Type IVB	Type I	Tra_I	*Pseudomonas putida* plasmid pGRT1	WP_014003986.1	WP_014003947.1	WP_014003944.1
205	Type IVB	Type I	Tra_I	*Salmonella enterica* subsp. enterica serovar Typhimurium plasmid R621a	WP_014065723.1	WP_001703917.1	WP_014065729.1
206	Type IVB	Type I	Tra_I	*Yersinia pseudotuberculosis* plasmid pGDT4	WP_012606356.1	WP_012606352.1	WP_012606350.1

					IcmO	DotA	IcmB
207	Type IVB	Type I	Dot/Icm	*Legionella pneumophila* subsp. *pneumophila* str. Philadelphia 1	WP_010946195.1	WP_010948386.1	WP_010946205.1
208	Type IVB	Type I	Dot/Icm	*Coxiella burnetii* CbuG_Q212	WP_012569739.1	WP_012569734.1	WP_012569743.1
					Tfc6	Tfc19	Tfc16
209	Type IVC (GI type)	Type G	Tfc	*Achromobacter xylosoxidans* A8	WP_013391804.1	WP_013391811.1	WP_013391807.1
210	Type IVC (GI type)	Type G	Tfc	*Acidovorax* sp. JS42	WP_011804677.1	WP_011804664.1	WP_011804669.1
211	Type IVC (GI type)	Type G	Tfc	*Aromatoleum aromaticum* EbN1	WP_011236068.1	WP_011236080.1	WP_011236077.1
212	Type IVC (GI type)	Type G	Tfc	*Bordetella petrii* DSM 12804	WP_012248333.1	WP_012248362.1	WP_012248346.1
213	Type IVC (GI type)	Type G	Tfc	*Cupriavidus metallidurans* CH34	WP_004350569.1	WP_004350620.1	WP_011516996.1
214	Type IVC (GI type)	Type G	Tfc	*Delftia acidovorans* SPH-1	WP_012205978.1	WP_008266205.1	WP_012205988.1
215	Type IVC (GI type)	Type G	Tfc	*Erwinia billingiae* Eb661	WP_013199775.1	WP_013199757.1	WP_013199764.1
216	Type IVC (GI type)	Type G	Tfc	*Gallibacterium anatis* UMN179	WP_013745179.1	WP_013745156.1	WP_013745159.1
217	Type IVC (GI type)	Type G	Tfc	*Haemophilus influenzae* plasmid ICEhin1056	WP_012564937.1	WP_011271852.1	WP_012564944.1
218	Type IVC (GI type)	Type G	Tfc	*Legionella pneumophila* str. Lens	WP_011215137.1	WP_011215140.1	WP_011213419.1
					PcfC	PrgH	PrgJ

219	Type IVD (Gram +)	Type GP	Prg/pcf	*Enterococcus faecalis* plasmid pBEE99	WP_002370353.1	WP_010815886.1	WP_002365863.1
220	Type IVD (Gram +)	Type GP	Prg/pcf	*Streptococcus agalactiae* NEM316	WP_000421159.1	WP_000651825.1	WP_011074733.1
					YdcQ	YddG	YddE
221	Type IVD (Gram +)	Type GP	Ydd	*Bacillus atrophaeus* 1942	WP_003328163.1	WP_003328178.1	WP_003328176.1
222	Type IVD (Gram +)	Type GP	Ydd	*Bacillus subtilis* subsp. *subtilis* str. 168	NP_388367.1	NP_388377.1	WP_009966629.1
223	Type IVD (Gram +)	Type GP	Ydd	*Staphylococcus aureus* subsp. aureus ED98	WP_001251209.1	WP_000681156.1	WP_001049264.1
					TcpA	TcpH	TcpF
224	Type IVD (Gram +)	Type GP	Tcp	*Clostridium perfringens CPE* str. F4969 plasmid pCPF4969	WP_011428886.1	WP_003470020.1	WP_003470014.1
225	Type IVD (Gram +)	Type GP	Tcp	*Clostridium perfringens* plasmid pCW3	WP_012478265.1	WP_003464372.1	WP_003479707.1
					Orf21_Tn	Orf15_Tn	Orf16_Tn
226	Type IVD (Gram +)	Type GP	Orf_Tn	*Clostridium difficile* 630	WP_011861922.1	WP_011861917.1	WP_011861918.1
227	Type IVD (Gram +)	Type GP	Orf_Tn	*Listeria monocytogenes* EGD-e	NP_464637.1	NP_464630.1	NP_464631.1
228	Type IVD (Gram +)	Type GP	Orf_Tn	*Streptococcus gallolyticus* UCN34	WP_000813488.1	WP_001574271.1	WP_000331160.1
229	Type IVD (Gram +)	Type GP	Orf_Tn	*Streptococcus suis* SC84	WP_000813488.1	WP_000804748.1	WP_000331160.1
